# miR-135a inhibits tumor metastasis and angiogenesis by targeting FAK pathway

**DOI:** 10.18632/oncotarget.16098

**Published:** 2017-03-10

**Authors:** Zhenguo Cheng, Funan Liu, Hongyan Zhang, Xiaodong Li, Yanshu Li, Jiabin Li, Furong Liu, Yu Cao, Liu Cao, Feng Li

**Affiliations:** ^1^ Department of Cell Biology, Key Laboratory of Cell Biology, Ministry of Public Health, and Key Laboratory of Medical Cell Biology, Ministry of Education, China Medical University, Shenyang 110122, China; ^2^ Department of Surgical Oncology, The first Hospital of China Medical University, Shenyang 110001, China

**Keywords:** gastric cancer, metastasis, miR-135a, FAK, p53

## Abstract

Tumor metastasis has been the major cause of recurrence and death in patients with gastric cancer. Here, we find miR-135a has a decreased expression in the metastatic cell lines compared with its parental cell lines by analyzing microRNA array. Further results show that miR-135a is downregulated in the majority of human gastric cancer tissues and cell lines. Decreased expression of miR-135a is associated with TNM stage and poor survival. Besides, regaining miR-135a in gastric cancer cells obviously inhibits tumor growth, migration, invasion and angiogenesis by targeting focal adhesion kinase (FAK) pathway. Bioinformatics analysis and molecular experiments further prove that miR-135a is a novel downstream gene of tumor suppressor p53. Blocking FAK with its inhibitor can also enhance miR-135a expression through inducing p53. In summary, this study reveals the expression and function of miR-135a in gastric cancer and uncovers a novel regulatory mechanism of miR-135a.

## INTRODUCTION

microRNAs are a class of small (19–24 nucleotides) non-coding RNAs which are involved in many biological processes, including cell proliferation, apoptosis and differentiation [1, 2]. During the past decades, accumulated studies have demonstrated that dysfunction of miRNAs plays a crucial role in the process of cancer proliferation, metabolism and metastasis [3]. Gastric cancer is common worldwide and has a high rate of metastasis. Although studies have confirmed that miRNAs play important roles in multiple steps of gastric cancer metastasis, such as angiogenesis, anoikis, epithelial mesenchymal transition and cancer stem cells [4, 5], the major miRNAs that control the metastasis of stomach cancer is still blur.

miR-135a functions as an oncogenic microRNA in colorectal carcinomas by repressing adenomatous polyposis coli (APC) [6]. Subsequently, studies demonstrate miR-135a contributes to the development of portal vein tumor thrombus in hepatocellular carcinoma [7] and leads to cervical cancer cell transformation through regulating SIAH1/β-catenin signaling [8]. While, some other studies find miR-135a is downregulated in pancreatic ductal adenocarcinoma [9], prostate cancer [10] and epithelial ovarian cancer [11]. Recovering the expression of miR-135a impairs the progression of breast and lung cancer by repressing EMT progress [12, 13] Recently, miRNA array analysis reveals that the expression of miR-135a is markedly decreased during lymphatic metastasis of gastric cancer. However, the function and regulatory mechanism of miR-135a in gastric cancer is ill defined.

By analyzing miRNA array, we find miR-135a is significantly arrested in the metastatic cell lines. Subsequently, lower expression of miR-135a in gastric cancer tissues is confirmed by real-time PCR assay, and the tumor suppressor functions for miR-135a are validated with clone formation, flow cytometry, transwell, tubule formation and tumor bearing nude mice assays. Our study proposes a novel FAK/p53/miR-135a loop signaling which may be a potential therapeutic target for metastatic gastric cancer.

## RESULTS

### miR-135a as a potential tumor metastasis suppressor is downregulated in gastric cancer

**Figure 1:**
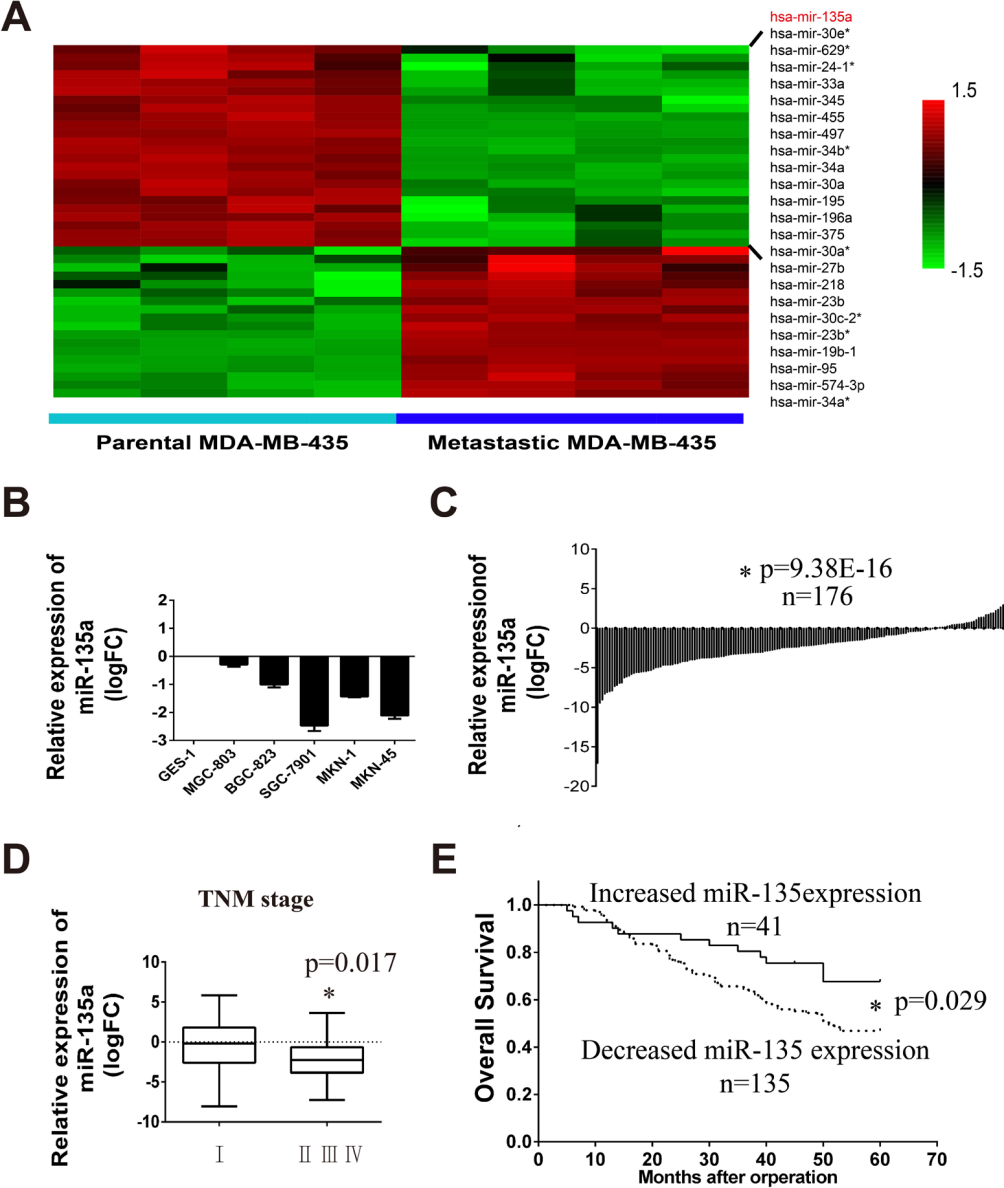
Tumor metastasis related miR-135a is downregulated in gastric cancer cell lines and tissues (**A**) Hierarchical clustering of decreased miRNAs in the lung metastasis MDA-MB-435 cells compared with the parent MDA-MB-435 cells. (**B**) Relative expression of miR-135a in five gastric cancer cell lines compared with normal gastric cell line GES-1, log2 fold change (logFC) was used. (**C**) Relative expression of miR-135a in 176 gastric cancer tissues compared with its corresponding para-cancer tissues. Value below zero baseline represents decreased expression in tumor tissues, and value above zero baseline represents increased expression. Independent sample *t-test* are used. (**D**) Relative expression of miR-135a in cancer tissues that have different pathologic stage status, One-Way ANOVA is used. (**E**) Kaplan-Meier survival analysis of 176 gastric cancer stratified by the status of miR-135a expression. “Increased miR-135a expression” represents patients that have an elevated miR-135a in tumor tissues compared with its corresponding para-cancer (logFC > 0); “Decreased miR-135a expression” represents patients that have lower miR-135a expression (logFC < 0). * *p <* 0.05

In order to identify metastasis related miRNAs, miRNA microarrays are retrieved in GEO database. Fortunately, a microarray data which contains MDA-MB-435 parental cells and lung metastasis MDA-MB-435 subline (GSE39358) is obtained. Differentially expression of miRNAs between them are analyzed with R package (change fold > 2 and *p* < 0.005) (Table 1). Figure 1A displays the hierarchical clustering of miRNAs in the parent and metastatic groups. 24 miRNAs which are significantly downregulated in lung metastasis cell lines are listed. From the results we can find miR-135a is decreased in the metastatic subline. So we wonder whether miR-135a is also a tumor suppressor in gastric cancer. Firstly, we examine the RNA level of miR-135a in 5 gastric cancer cell lines (MGC-803, BGC-823, SGC-7901, MKN1 and MKN45) and one normal gastric cell line (GES-1) by Real-time PCR assay. As shown in Figure 1B, miR-135a level is obviously decreased in 5 gastric cancer cell lines. Afterwards, we assess the expression of miR-135a in 176 pairs of gastric cancer tissue and its corresponding para-cancer tissues collecting from the First Affiliated Hospital of China Medical University (details are listed in Supplementary Table 1). Figure 1C shows that the majority of tumor tissues (135/176) has a lower miR-135 level than its corresponding normal tissues. Further analysis reveals that cancer tissues in advanced TNM stages possess a lower level of miR-135a compared with the early stage ones (Figure 1D). The effect of miR-135a expression on gastric cancer prognosis is also examined by constructing Kaplan-Meier curves and difference between groups is compared by Log-rank test. Results show that patients with increased miR-135a (41/176) have a better overall survival, suggesting miR-135a may be a prognosis factor of gastric cancer (Figure 1E).

**Table 1:** Differential expression of miRNAs between metastasis cell lines and parent cell lines

miRNAs name	Change fold
**miRNAs upregulated (metastasis vs parent)**	
hsa-miR-652	2.02
hsa-miR-106b	2.1
hsa-miR-106b*	2.91
hsa-miR-1274a	2.18
hsa-miR-1275	2.39
hsa-miR-21*	2.37
hsa-miR-25	2.52
hsa-miR-25*	2.5
hsa-miR-26a-1*	11.89
hsa-miR-302a	7.87
hsa-miR-330-3p	145.2
hsa-miR-374a	116.81
hsa-miR-421	2.1
hsa-miR-489	15.38
hsa-miR-550*	2.56
hsa-miR-630	4.08
hsa-miR-652	2.15
hsa-miR-93	2.19
**miRNAs downregulated (metastasis vs parent)**	
hsa-miR-196a	0.11
hsa-miR-135a	0.37
hsa-miR-195	0.35
hsa-miR-19b-1*	0.35
hsa-miR-218	0.21
hsa-miR-23b	0.41
hsa-miR-23b*	0.39
hsa-miR-24-1*	0.32
hsa-miR-27b	0.35
hsa-miR-30a	0.19
hsa-miR-30a*	0.15
hsa-miR-30c-2*	0.36
hsa-miR-30e*	0.45
hsa-miR-33a	0.44
hsa-miR-345	0.49
hsa-miR-34a	0.28
hsa-miR-34a*	0.19
hsa-miR-34b*	0.34
hsa-miR-375	0.42
hsa-miR-455-3p	0.27
hsa-miR-497	0.27
hsa-miR-574-3p	0.22
hsa-miR-629*	0.33
hsa-miR-95	0.24

### FAK is a novel target of miR-135a in gastric cancer

**Figure 2:**
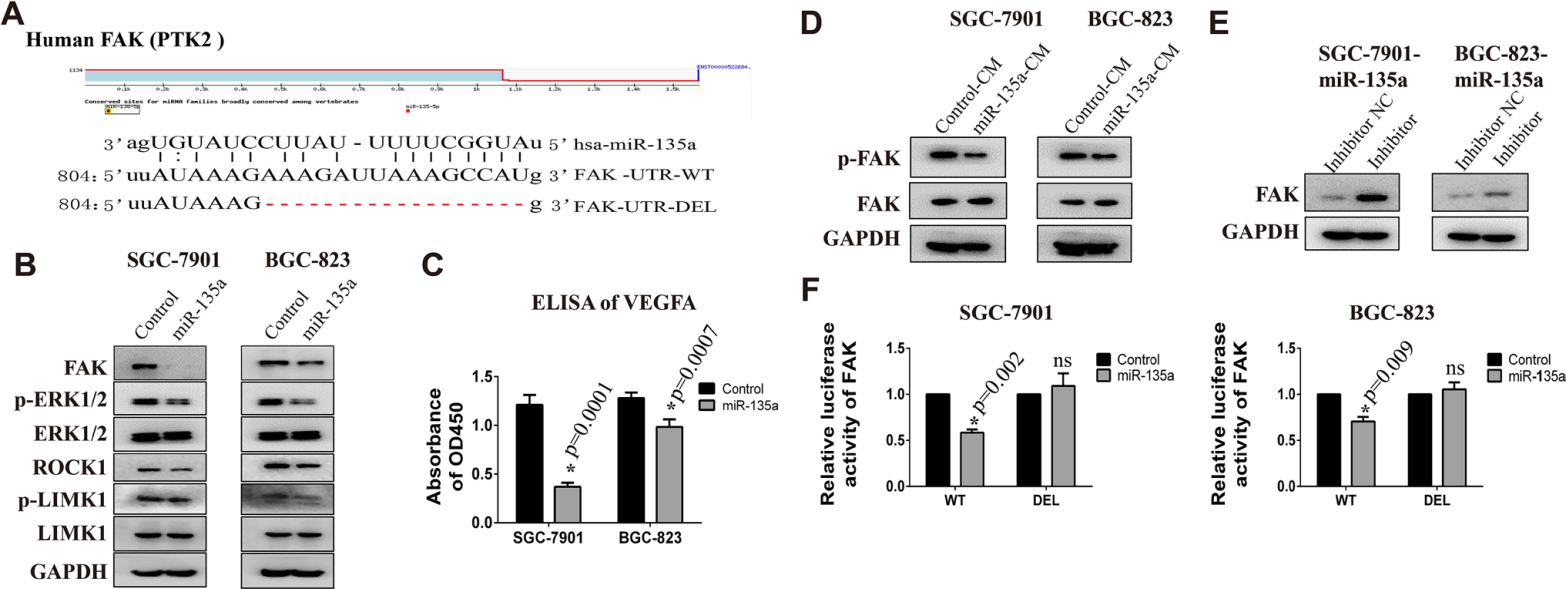
FAK is a target gene of miR-135a (**A**) The binding site of miR-135a in the FAK 3′UTR and the skeleton of FAK 3′UTR luciferase reporter vector. (**B**) The protein expression of FAK, p-ERK, ERK, ROCK1, p-LIMK1 and LIMK1 in SGC-7901 and BGC-823 stable cell lines are examined by Western Blot. (**C**) Relative expression of secreted VEGFA in the culture supernatant of cancer cells is detected by ELISA assay. (**D**) The protein expression of phosphorylated FAK (pTyr397) in cells that treated with conditioned medium from control or miR-135a overexpressing stable cell lines. (**E**) miR-135a overexpressing cells were transfected with 200 nM miRNA inhibitor or control RNA, then FAK protein level was examined with Western blot assay. (**F**) The effect of miR-135a mimics on the luciferase activity of FAK 3′UTR wild type (WT) and deletion type (DEL) are detected by luciferase reporter assay. * *p <* 0.05

To clarify the mechanism of miR-135a in tumor metastasis, potential target genes of miR-135a are predicted and the functional enrichment analysis of these genes are analyzed with StarBase software. As shown in Table 2, 17 pathways are identified. As angiogenesis is a hallmark of cancer and has been identified as a crucial component of cancer progression and distant organ metastasis. Moreover, miR-135a is markedly decreased in the metastatic MDA-MB-435 subline which is isolated from lung metastasis, indicating angiogenesis may be crucial target pathway of miR-135a. During the last decades, extensive studies in cultured cells as well as conditional FAK knockout mice modes indicate a critical role of FAK in angiogenesis during cancer progression [14]. In addition, FAK is also an important regulator and effector of VEGF in tumor angiogenesis. So we focus our attention on FAK in this study. Potential miRNAs binding sites of FAK are predicted with TargetScan and microRNA.org software. Figure 2A displays miR-135a binding site on the 3′UTR of FAK. Our precious studies have found SGC-7901 and BGC-823 have strong metastatic capability, so these two cells are used to evaluate the function of miR-135a. Firstly, we construct miR-135a overexpressing cell lines by infecting with lentivirus, and the infection efficiency is validated by Real-time PCR (Supplementary Figure 1A). We next investigate the protein expression of FAK in stable cell lines with western blot assay. As shown in Figure 2B, regaining miR-135a significantly inhibits the protein expression of FAK. Previous study has proved that FAK can facilitate angiogenesis by activating MAPK/VEGFA pathway [15]. Then we detect the level of phosphorylated ERK1/2 and VEGFA with western blot and ELISA assays respectively. As expected, the expression of phosphorylated ERK1/2 (Figure 2B) and VEGFA (Figure 2C) are declined in miR-135a overexpressing cells. Our data also shows miR-135a can slightly suppress another FAK associated pathway ROCK1/LIMK1 which has been proved a target of miR-135a in prostate recently [10].

**Table 2:** The functional cluster of miR-135a interacted target genes

PANTHER Pathway Name	Hit Genes	*P* value
EGF receptor signaling pathway	CBLB,MAP2K4,NF1,PHLDB2,PIK3CD,PRKD3,RASAL2,STAT6,YWHAG	0.000161876
Oxidative stress response	DUSP5,ELK1,MAP2K4,MEF2A, MEF2C	0.00126669
Insulin/IGF pathway-protein kinase B signaling cascade	GSK3B,IRS2,PIK3CD,PIK3R2	0.00284108
p38 MAPK pathway	ELK1,MAP2K4,MEF2A,MEF2C	0.00284108
Hedgehog signaling pathway	CSNK1A1,GSK3B,SUFU	0.00390938
Alpha adrenergic receptor signaling pathway	PLCB1,STX6,VAMP2	0.0053178
Angiogenesis	FZD1,GSK3B,MAP2K4,PIK3CD,PIK3R2,PRKD3,PTK2,TCF7L2	0.00578641
TGF-beta signaling pathway	ACVR1B,BMPR2,FKBP1A,SKI, SMAD5,SMURF2	0.00583315
Interleukin signaling pathway	ELK1,ELK3,ELK4,GSK3B,IRS2, STAT6	0.00583315
PDGF signaling pathway	ELK1,ELK4,GSK3B,PIK3CD, PIK3R2,RPS6KB1,STAT6	0.00737287
Ras Pathway	ELK1,GSK3B,MAP2K4,PIK3CD, RGL1	0.007719
Wnt signaling pathway	ACVR1B,CSNK1A1,CSNK1G2,CSNK1G3,FZD1,GSK3B,PLCB1,SIAH1,SMAD5,SMARCE1,TCF7L2	0.00788754
PI3 kinase pathway	FOXO1,GSK3B,PIK3R2,RPS6KB1	0.0101668
p53 pathway	KAT6B,PIK3CD,PIK3R2,SIAH1, SIRT1	0.0142272
Parkinson disease	CSNK1A1,CSNK1G2,CSNK1G3, ELK1, YWHAG	0.0189871
VEGF signaling pathway	PIK3CD,PIK3R2,PRKD3,PTK2	0.0195784
Angiotensin-II-stimulated signaling through G proteins	ELK1,GRK5,PLCB1	0.0231895

Studies have demonstrated that VEGF could promote tumor angiogenesis by activating FAK [16]. Therefore, we assess the activated FAK in gastric cancer cells that treated with conditioned medium (CM) from control or miR-135a overexpressing cells. As shown in Figure 2D, cells treated with CM from miR-135a overexpressing cells has a lower expression of p-FAK, indicating miR-135a can also repressed VEGF-mediated FAK activation. To further confirm FAK is a target of miR-135a, miRNA inhibitors are transfected into overexpressing cells to weak miR-135a effect. As expected, the expression of FAK recovered when cells treated with inhibitor (Figure 2E).

To investigate the direct binding of miR-135a to FAK 3′UTR, luciferase reporter assay is performed. Figure 2E shows that co-transfection miR-135a mimics together with luciferase plasmids reduces the luciferase activity of WT FAK-3′UTR. While, this effect is blocked when co-transfected with binding-site-deletion luciferase plasmid (DEL FAK-3′UTR). All these results prove miR-135a is able to inhibit the translation of FAK by binding to its 3′UTR.

### miR-135a inhibits gastric cancer growth and tubule formation by targeting FAK

**Figure 3:**
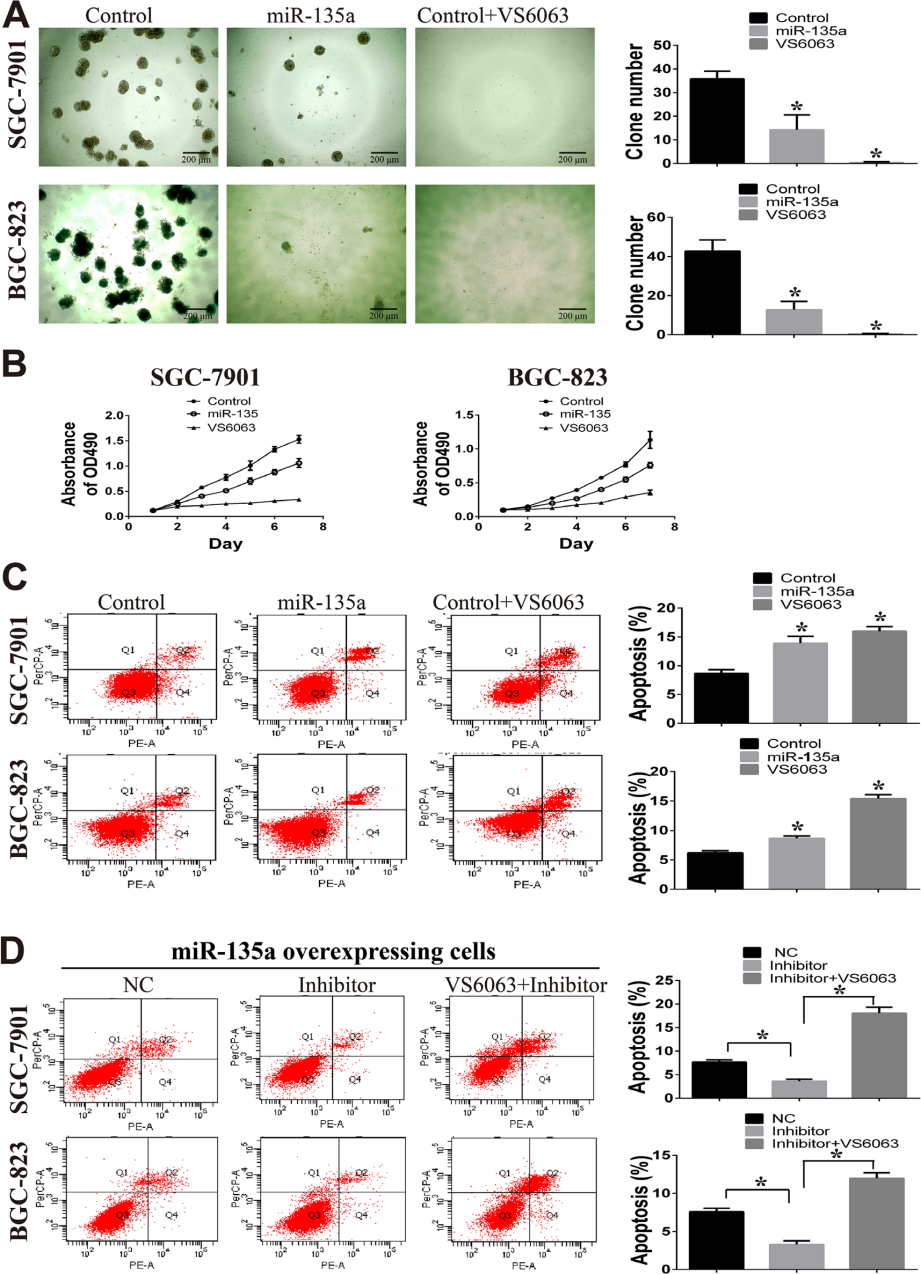
miR-135a suppress cell growth *in vitro* (**A**) Clone formation assay is used to examine the effect of miR-135a and FAK inhibitor (VS6063, 10μM) on two gastric cancer cells. All cells are cultured in matrigel for 15 days. (**B**) The effect of miR-135a and FAK inhibitor (VS6063, 10μM) on cell viability are examined by MTT assay. (**C**) Apoptotic cells in miR-135 overexpressing and VS6063-treated cells are analyzed with Annexin V-PE and 7-amino-actinomycinD. (**D**) miR-135a overexpressing cells are treated with nonsense control RNA (NC), miRNA inhibitor (200nM) or miRNA inhibitor (200nM) in combination with VS6063 (10μM), then apoptotic cells are analyzed with Annexin V-PE and 7-amino-actinomycinD. All data are analyzed with GraphPad Prism, and paired sample *t-test* is used, * *p <* 0.05.

To disclose the effect of miR-135a on tumor growth, clone formation in matrigel and MTT assays are performed. As shown in Figure 3A, clone numbers in miR-135a overexpressing groups are fewer than control groups. Moreover, there are hardly any clones when control cells are treated with 10 μM FAK inhibitor VS6063. Consistent with these findings, we observe marked suppression of cell viability in miR-135a overexpressing and VS6063-treated cells by performing MTT assay (Figure 3B).

Considering FAK is known to inhibit tumor growth by inducing apoptosis, so we wonder whether the effect of miR-135a on cell growth is caused by apoptosis. Flow cytometry analysis shows that the proportion of apoptotic cells is increased in SGC-7901 and BGC-823 cells with forced expression of miR-135a (Figure 3C). Similar effect on apoptosis is observed when FAK is blocked with inhibitor VS6063. To further confirm the effect of miR-135a/FAK axis on cell apoptosis, miRNA inhibitors and FAK inhibitors are used. As shown in Figure 3D, when blocking miR-135a with miRNA inhibitors, apoptotic cells in miR-135a overexpressing cells are decreased. However, reverse result is observed in cells treated with miRNA inhibitor in combination with VS6063, suggesting the suppression of cell apoptosis is in a FAK-depend manner.

**Figure 4:**
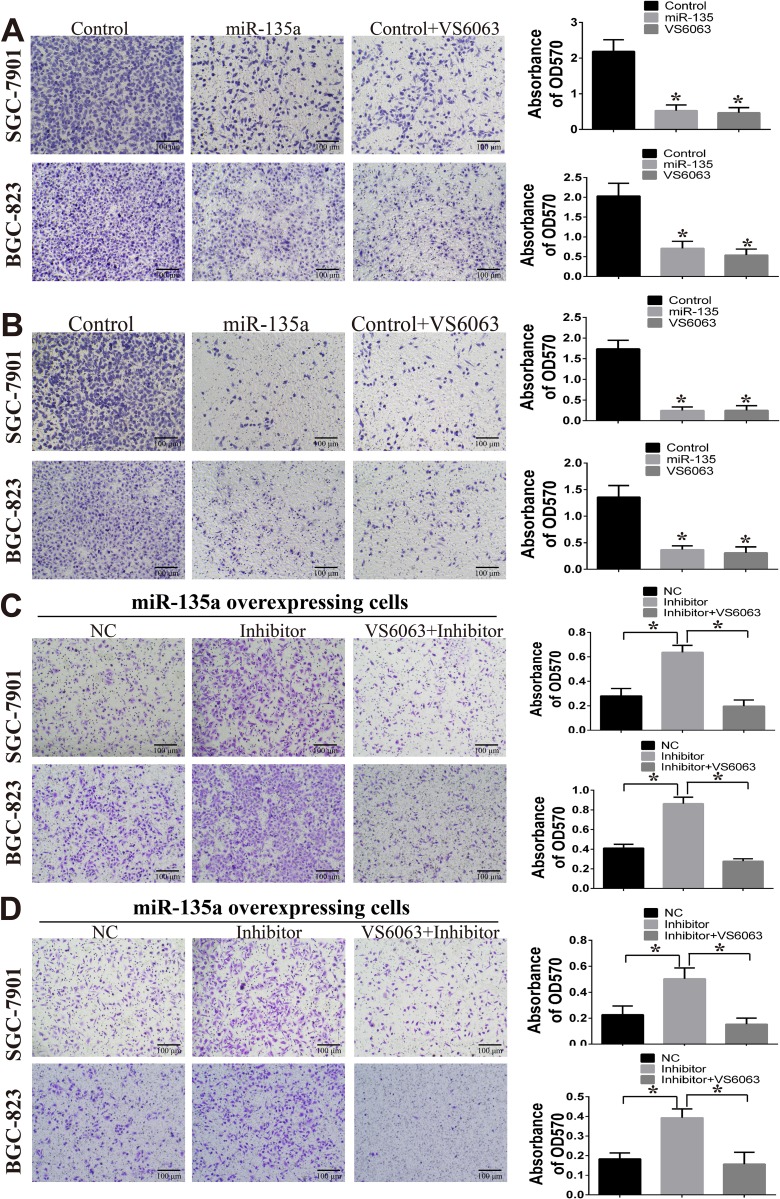
miR-135a inhibits cell migration and invasion (**A**) The effect of miR-135a and FAK inhibitor (VS6063) on cell migration are examined by transwell assay (200×). (**B**) The effect of miR-135a and FAK inhibitor (VS6063) on cell invasion are examined by transwell assay (200×). (**C**) miR-135a overexpressing cells are treated with nonsense control RNA (NC), miRNA inhibitor (200 nM) or miRNA inhibitor (200 nM) in combination with VS6063 (10 μM), then migration assay is performed. (**D**) miR-135a overexpressing cells are treated with miRNA inhibitor (200 nM) or miRNA inhibitor (200 nM) in combination with VS6063 (10 μM), then invasion assay is performed. For A & B, cells are firstly seeded into 6-wells, then treated with FAK inhibitor for 24 h, washed with PBS and re-seeded into transwell insert. For C& D, cells are firstly transfected with 200 nM nonsense control RNA (NC) or miRNA inhibitor (200 nM) for 24 h, then treated with FAK inhibitor for 24 h, washed with PBS and re-seeded into transwell insert

Next, transwell assay is performed to investigate the effect of miR-135a on cell migration and invasion. As shown in Figure 4A, fewer migrated miR-135a overexpressing cells and VS6063-treated cells are seen on the insert membrane than control cells. This difference of cell numbers among groups are also estimated by measuring the OD570 value of the eluted crystal violet stain (right panel). Similar results are observed in the invasion assay (Figure 4B). To further examine the inhibitory effect of miR-135a on cell migration and invasion is caused by targeting FAK, miRNA inhibitors and FAK inhibitor are used. After blocking miR-135a with miRNA inhibitor, the migration and invasion ability of miR-135a overexpressing cells are enhanced. However, this change is reversed when miRNA inhibitors are co-administered with FAK inhibitor VS6063 (Figure 4C and 4D).

**Figure 5:**
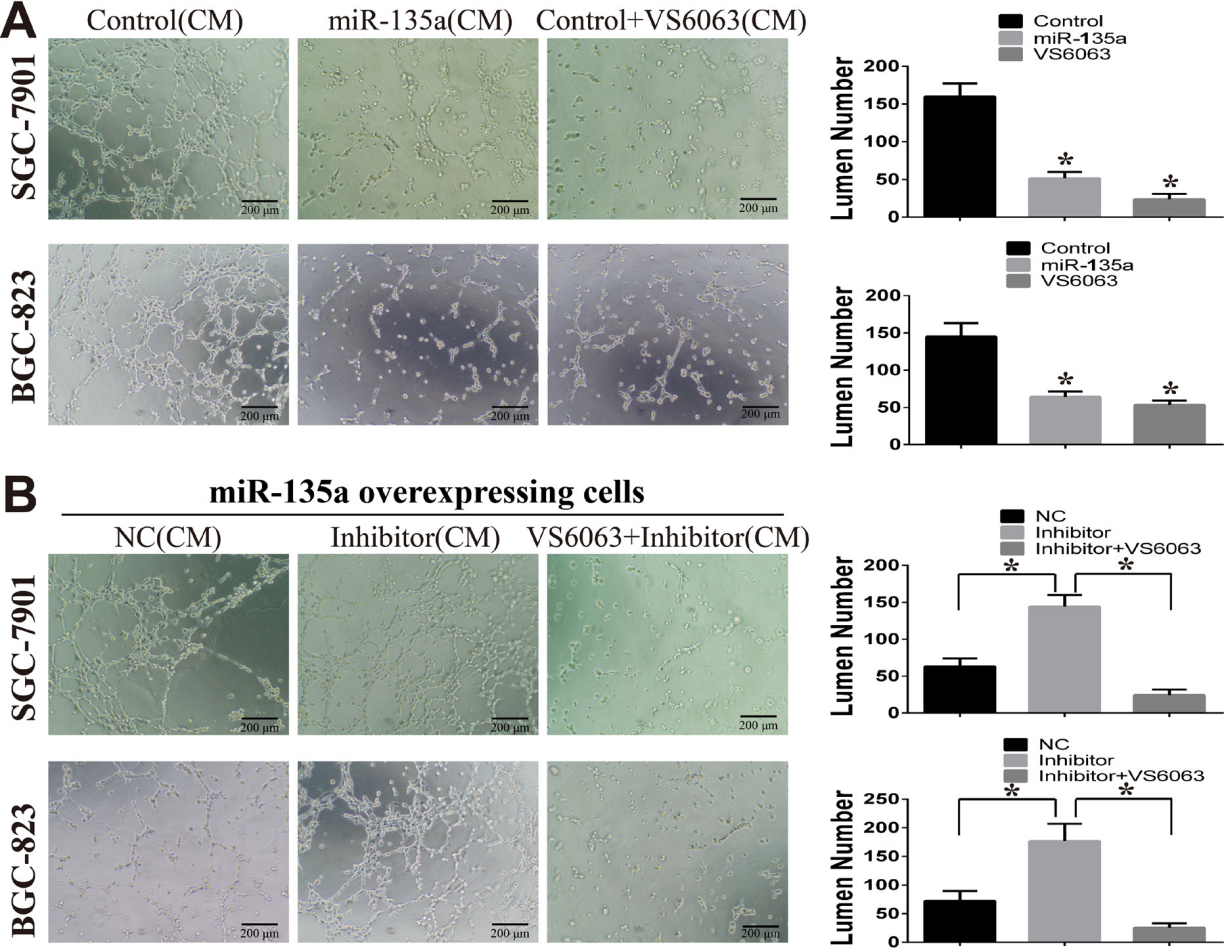
miR-135a suppress tubules formation of HUVEC cells (**A**) The effect of miR-135a and FAK inhibitor (VS6063) on HUVEC tubules formation is assessed by tubule formation assay. Conditioned medium (CM) from stable cells lines or VS6063-treated cells are used. (**B**) miR-135a overexpressing cells are treated with nonsense control RNA (NC), miRNA inhibitor (200nM) or miRNA inhibitor (200 nM) in combination with VS6063 (10 μM), then CM are collected and used for tubules formation. All data are analyzed with GraphPad Prism, and paired sample *t-test* is used, * *p <* 0.05.

Large number of studies have demonstrated FAK is an important pro-angiogenic gene in many tumors [17, 18]. Our above results show that miR-135a inhibits the production of VEGF and activation of FAK. To evaluate whether miR-135a can inhibit cancer angiogenesis by targeting FAK/VEGF pathway, cell tubules formation of HUVEC cells are evaluated. HUVEC cells that stimulated with conditioned medium (CM) from miR-135a overexpressing cells or VS6063 pre-treated cells, have a decreased tubules formatting ability compared with the control groups (Figure 5A). While, when miR-135a expression is blocked with its inhibitor, the tubules formation is enhanced. Strikingly, HUVEC cells stimulated with CM from miRNA inhibitor in combination with VS6063 group have fewer tubules (Figure 5B).

### miR-135a inhibits tumor growth and angiogenesis in nude mice

**Figure 6:**
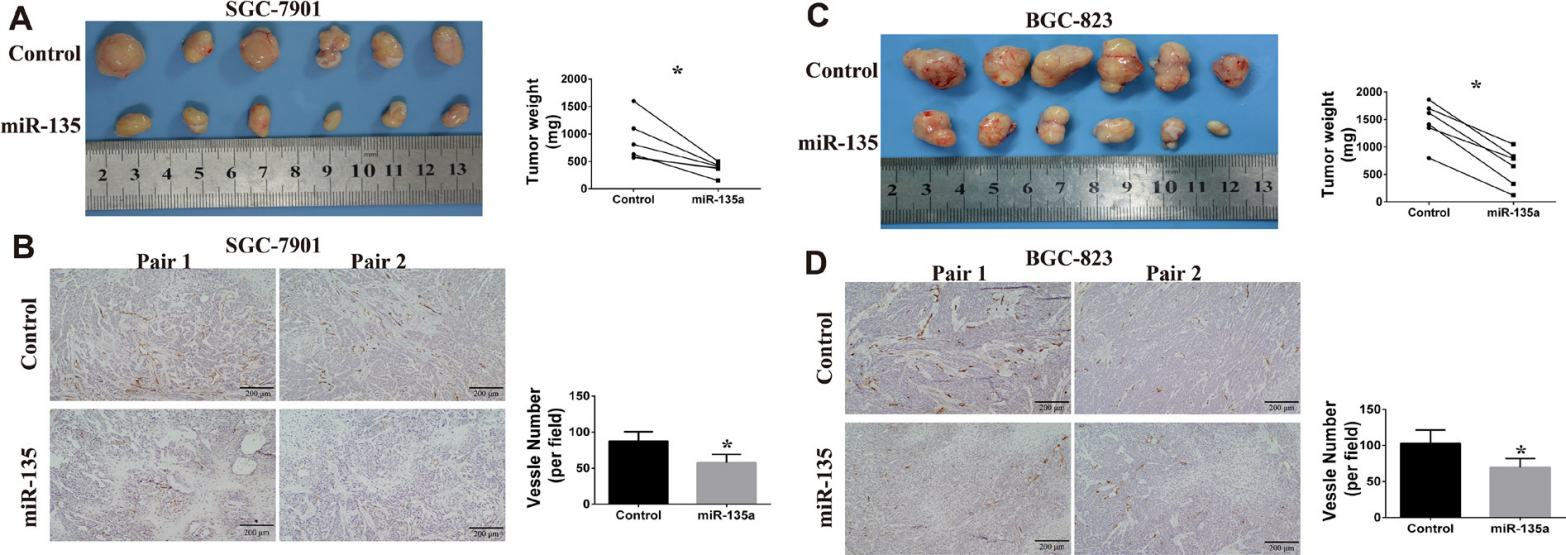
Effects of miR-135a on tumor growth and angiogenesis *in vivo* (**A**) Xenografts tumors from SGC-7901 are photographed, and tumor weight are measured with electronic balance. (**B**) Angiogenesis in xenografts tumors from SGC-7901 are evaluated by CD34 immunohistochemistry (100×). The expression of CD34 in SGC-7901 tumor sections is examined by immunohistochemistry (100×). (**C**) Xenografts tumors from BGC-823 are photographed, and tumor weight are measured with electronic balance. (**D**) Angiogenesis in xenografts tumors from BGC-823 are evaluated by CD34 immunohistochemistry (100×). Blood vessel number is analyzed with Image Pro Plus software.

Our previous study has confirmed gastric cancer cells that injected in the armpits has capability of spontaneous metastasis [19]. To determine the anti-tumor function of miR-135a, we inject control and miR-135a overexpressing cells into the bilateral axillary of BALB/c nude mice respectively. As shown in Figure 6A, miR-135a overexpressing SGC-7901 has a definitely smaller tumor volume than control group. To evaluate the effect of miR-135a on angiogenesis, tumor sections from nude mice are stained with CD34 and hematoxylin. Results of immunohistochemistry show that fewer CD34 positive expression is obtained in miR-135a overexpressing group (Figure 6B). We also assess the effect of miR-135a in BGC-823 cell lines. Similarly, regaining miR-135a is able to inhibit tumor growth and blood vessels formatting in BGC-823 cell lines (Figure 6C and 6D). Taken together, these findings indicate that miR-135a is a tumor suppressor in gastric cancer by influencing tumor growth and angiogenesis.

### FAK is upregulated and negatively associated with miR-135a in tumor tissues

**Figure 7:**
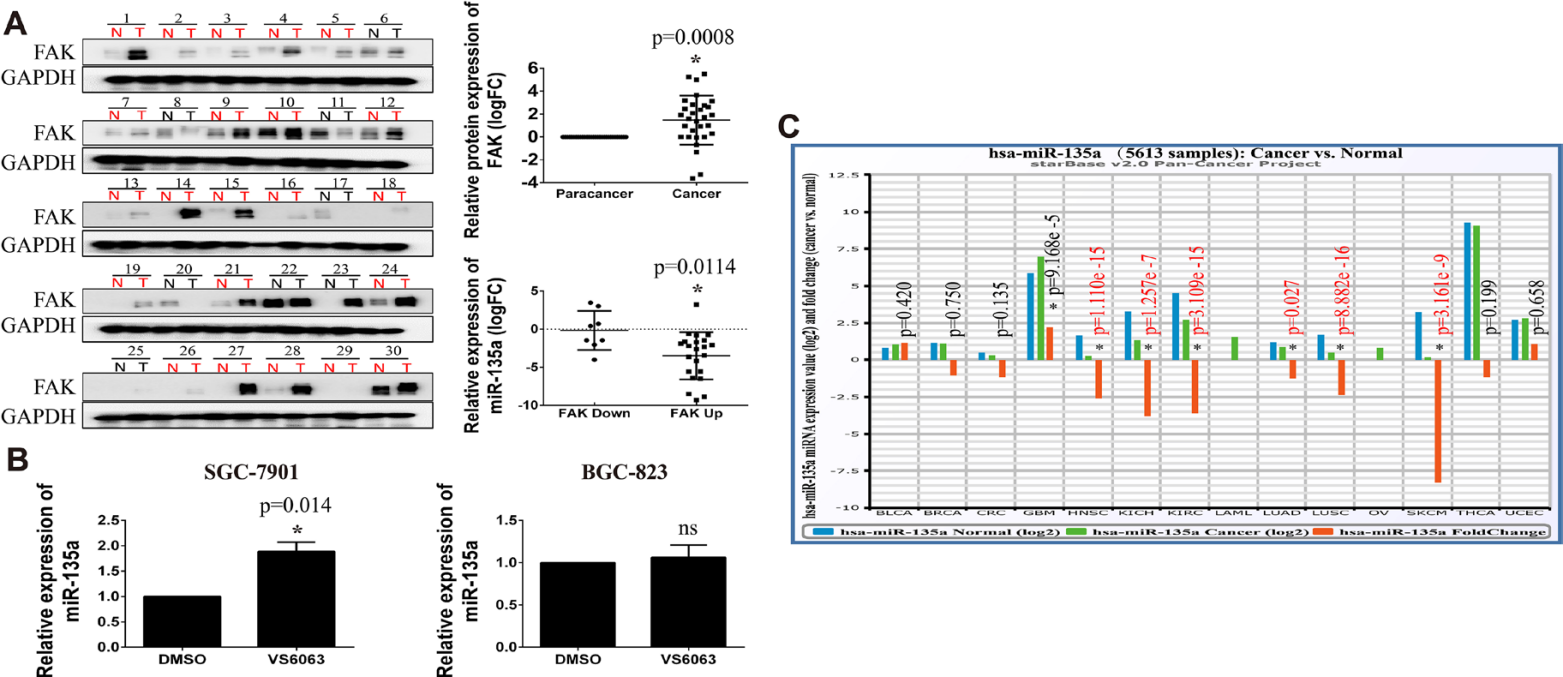
FAK is upregulated in gastric cancer and negatively correlated with miR-135a (**A**) The protein expression of FAK in 30 gastric tissues is detected by Western Blot assay, the relative expression is analyzed with Image J software and the expression of miR-135a in different FAK status is analyzed by GraphPad Prism with student *t* test. (**B**) Relative expression of miR-135a in VS6063-treated gastric cells is detected with Real-time PCR. (**C**) The expression of miR-135a in other tumors is analyzed with StarBase software.

It is reported that FAK is overexpressed in gastric cancer, and elevated expression of FAK is significantly correlated with cancer progression and poor prognosis [20]. In this paper, we examine the protein expression of FAK in 30 gastric cancer tissues. As shown in Figure 7A, FAK protein level in cancer tissues is markedly elevated compared with its para-cancer tissues (*p* = 0.0048). Further analysis reveals that patients with increased FAK (FAK up, Cancer vs. Para-cancer) has a lower expression of miR-135a than patients with decreased FAK (FAK down) (*p* = 0.0114). However, this finding does not exclude that FAK may be a potential upstream gene of miR-135a. So the expression of miR-135a in VS6063-treated cells are examined with Real-time PCR assay. Surprisingly, elevated miR-135a level is observed in SGC-7901 cells, but no statistically significant changes were noted in BGC-823 cells (Figure 7B), hinting the regulation of miR-135a by FAK may be dependent on cell context. Previous studies have shown miR-135a also acts as an oncogene in some carcinomas. To better understand the function of miR-135a in cancer, we analyzes the expression of miR-135a in other cancer tissues with Starbase software. Interestingly, miR-135a is decreased in head & neck, lung, kidney, skin cancers, while increased in glioblastoma multiforme (GBM) (Figure 7C). These findings indicate distinct function of miR-135a varies among tumors depending on the landscape of tissue types.

### miR-135a is a target gene of p53 in gastric cancer

**Figure 8:**
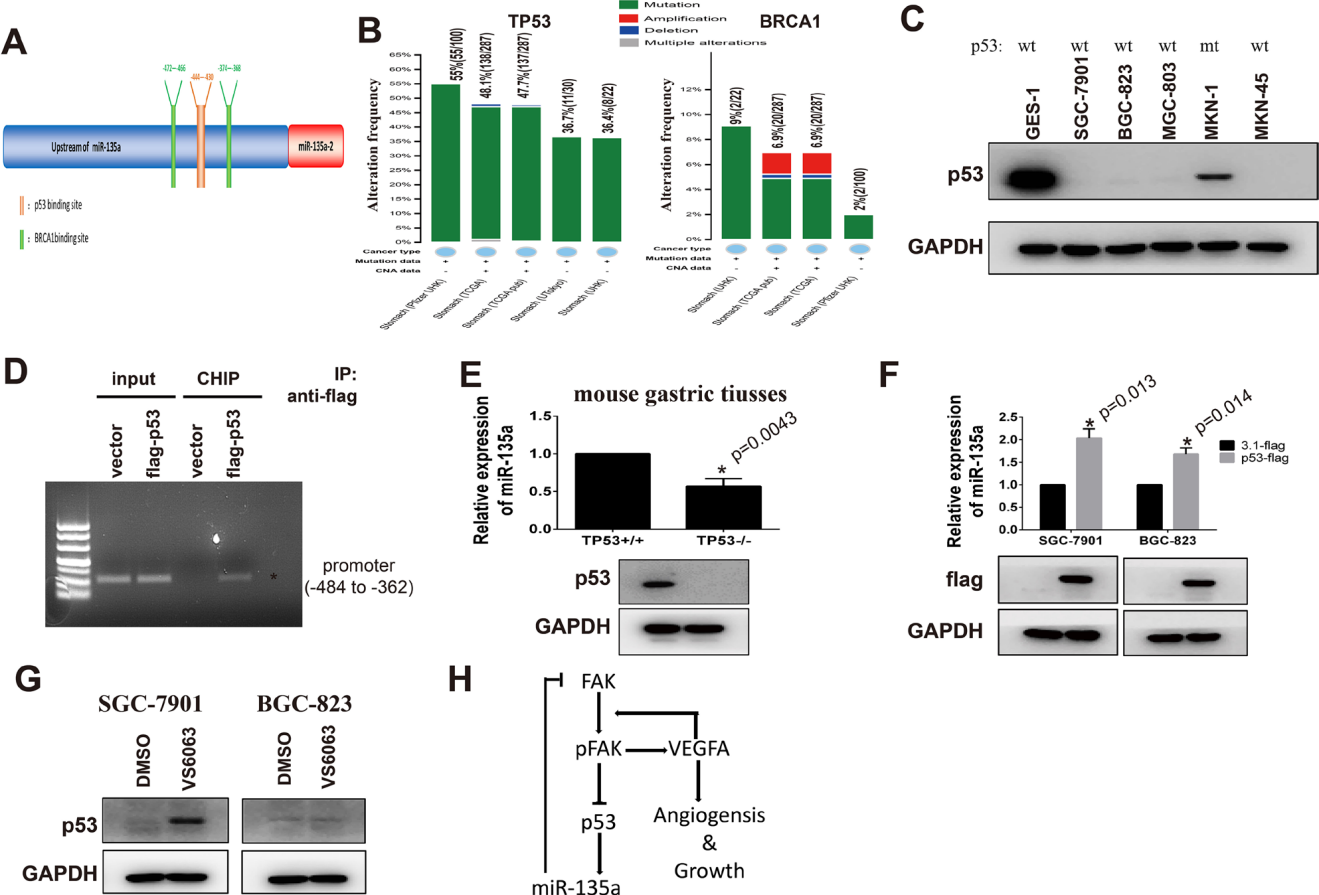
miR-135a is a target gene of p53 in gastric cancer (**A**) Bioinformatics analysis of miR-135a promoter with Jaspar software. (**B**) The gene status of TP53 and BRCA1 in gastric cancer is analyzed with cBioPortal software. (**C**) The expression p53 in gastric cancer cell lines is examined by Western bot, and mutant status are confirmed by PCR sequencing. (**D**) p53- DNA complex in gastric cancer cells is isolated by Chromatin immunoprecipitation, and specific p53-binding site on miR-135a promoter (-484 to -362) is identified by PCR. (**E**) Relative expression of miR-135a in gastric tissues from p53 knocking out (p53^−/−^) or wild type mice (p53^+/+^) is examined by Real-time PCR, and p53 protein level is validated by Western Blot. (**F**) Exogenous p53 is transfected into gastric cancer cells and relative expression of miR-135a is examined by PCR, transfection efficiency is confirmed by Western blot. (**G**) Gastric cancer cells are treated with 10 μM VS6063, then p53 protein level is examined by Western blot. (**H**) Model of FAK/p53/miR-135a loop signaling.

In order to identify the potential regulators of miR-135a, promoter of miR-135a is analyzed with Jaspar software (75% threshold), and the potential transcriptional factors (TFs) are listed in Supplementary Table 2. As important tumor suppressors, *p*53 and BRCA1 modulate the transcription of various downstream targets. We can also find that there are two BRCA1 binding sites and one *p*53 binding site on the promoter of miR-135a (Figure 8A). Numerous studies declare mutant of p53 or BRCA1 markedly impair their transcriptional activity, then we analyze the gene status of them in gastric cancer with Cbioportal. Results show the mutation rate of p53 up 55% in 100 tissues from HongKong and 48% in 287 tissues from TCGA, while the mutation rate of BRCA1 is less than 10% (Figure 8B). It is reported that BRCA1 can regulate gene transcription by interacting with p53 [21, 22]. Our results in protein-protein interaction analysis also display p53 can interact with BRCA1 (Supplementary Figure 1B). All findings indicate p53 may be the major regulator of miR-135a in gastric cancer.

Our above data has proved that miR-135a is decreased in 5 gastric cancer cells. To investigate whether p53 is a regulator of miR-135a, we firstly measure the levels of p53 in gastric cancer cell lines by Western blot. As shown in Figure 8C, the expression of p53 in p53 wild-type (wt) cancer cells including SGC-7901, BGC-823, MGC-803 and MKN-45 is lower than GES-1 cells. Although p53 is detectable in MKN-1 cells, it is reported that p53 in this cell lines is mutant. CHIP assay analysis validates that p53 can bind to the (-484 to -362) region of the promoter which contains both two BRCA1 bind sites and the p53 binding site (Figure 8D). To further confirm miR-135a is a downstream target of p53, miR-135a levels in primary gastric tissues from p53 wild type (p53^+/+^) and knocking out (p53^−/−^) mice are examined by Real-time PCR assay. Figure 8E shows p53-lacking gastric tissues have a lower expression of miR-135a than control tissues. Inversely, when we elevate p53 by transfecting wild p53 plasmid, the expression of miR-135a is partly restored (Figure 8F). Study has reported that FAK is involved in the degradation of p53. So we examine the effects of VS6063 on p53 expression with Western blot. As presented in Figure 8G, blocking FAK induces p53 in SGC-7901 cells, but do not affect the expression of p53 in BGC-823 cells. These findings are consistent with the above results in which increased miR-135a expression is only appeared in SGC-7901 cells.

## DISCUSSION

Tumor metastasis is a crucial hallmark of cancer progression, and the major cause of cancer morbidity and mortality [23]. Although a remarkable progress has been made in understanding the molecular mechanism of metastasis during the last decades, there are still a lot of questions remain faint. In this study, we demonstrate that miR-135a is a crucial regulator of tumor metastasis. Regaining the expression of miR-135a in gastric cancer cells can inhibit tumor growth, invasion and angiogenesis by targeting FAK/VEGF pathway. Besides, our data identify miR-135a as a novel target gene of p53, and can form a loop signaling with FAK (Figure 8H).

miRNA is a kind of crucial molecule which is associated with numerous cellular processes and also identified as critical regulator in cancer initiation and metastatic progression [24]. Some miRNAs including miR-206, miR-34a, miR-335, miR-141 are decreased in gastric tumor tissue and are associated with liver or lymphoma node metastasis [25–28]. However, miRNAs such as miR-199, miR-223, miR-107 and miR-372 are increased and promote liver or lymphoma node metastasis [29–32]. In this study, we find miR-135a is significantly decreased in metastasis cell lines compared with the parent lung metastasis cell lines, indicating miR-135a may be a key tumor metastasis suppressor.

It is known that miR-135a can function as a selective killer of malignant glioma by targeting STAT6, SMAD5 and BMPR2 [33]. In ovarian cancer, miR-135a inhibits the growth and survival of cancer cells by repressing HOXA10 [11]. Besides, low miR-135a expression often has a higher probability of relapse and a shorter disease free survival in hodgkin lymphoma. Our results demonstrate that the expression of miR-135a is markedly reduced in gastric cancer cell lines and tissues. Low miR-135a expression is associated with advanced tumor TNM stage and poor patients′ survival. By analyzing potential target genes, we find miR-135a is involved in various signaling pathways including angiogenesis, VEGFA, EGF, oxidative stress response, p38, TGFβ and interleukin pathway. Further experiments *in vitro* reveal miR-135a can targeting FAK/ERK/VEGFA and ROCK/LIMK pathway. Recovering miR-135a in gastric cancer cells obviously represses the cell growth, migration, invasion, HUVEC cells tubule formation *in vitro*, and impairs tumor growth and angiogenesis in nude mice. Moreover, we also find miR-135a is decreased in head & neck, lung, kidney, skin cancers, while it is increased in glioblastoma multiforme, hinting opposite function of miR-135a is determined by different genetic context.

Focal adhesion kinase (FAK), also known as protein tyrosine kinase 2 (PTK2), is a nonreceptor tyrosine kinase which plays a crucial role in regulating cell adhesion, proliferation, apoptosis, migration, invasion and angiogenesis [34]. Massive studies have proved FAK is hyperactivated and overexpressed in various tumors including lung, pancreatic, stomach, colorectal, head and neck, thyroid and breast carcinoma [35]. Many genes can promote the secretion of VEGFA in cancer cells by controlling FAK expression or activity, then activates FAK pathway in endothelial cells, resulting tumor angiogenesis [36, 37]. Moreover, FAK selective inhibitor such as VS6063 (Defactinib) and PND-1186 have entered clinical trials for carcinoma therapy [38]. Recently, studies prove some miRNAs including miR-7, miR-23b and miR-138 can inhibit tumor metastasis by targeting FAK [39–41]. In this study we find that FAK is overexpressed in gastric cancer tissues. Blocking the activity of FAK with VS6063 significantly inhibits tumor growth and tubule formation. Our data also indicates FAK can inhibit the expression of miR-135a by downregulating p53.

P53 is one of the most important tumor suppressor which plays an important role in maintaining genomic stability and regulating the cell cycle. High mutation rate of p53 gene has been found in many human cancers, making p53 one of the most studied gene in tumor [42]. Accumulation of mutated p53 is associated with the proliferation, drug resistance and metastasis of many types of human cancer [43]. In gastric cancer, p53 mutation is closely related with tumor differentiation, metastasis stage, vessel invasion, lymph node metastasis and poor prognosis [44, 45]. Wild type p53 can inhibit cancer chemoresistance, migration, metabolism and stemness by promoting expression of miR-34a [46]. While, mutated p53 leads to chemoresistance, and metastasis of cancer by upregulating onco-miRNAs such as miR-182 and miR-320c/d [47, 48]. By analyzing whole genomic sequencing data, we find p53 is mutated in most gastric tumor tissues. Further experiments reveal that p53 can bind the promoter of miR-135a. Stomach tissues in p53 knocking out mice have a lower miR-135a expression compared with p53 wild type. Regaining p53 in gastric cancer promotes miR-135a expression, indicating p53 mutant may be a reason for the downregulation of miR-135a in gastric cancer.

Taken together, this study shows that miR-135a is a key metastasis related miRNA and significantly downregulated in gastric cancer. Overexpression of miR-135a markedly inhibits the growth, migration and angiogenesis of gastric cancer by impairing FAK pathway. Our data also demonstrate miR-135a is a novel target of p53, and can form a FAK/p53/miR-135a loop signaling, making it a potential therapeutic target for metastatic gastric cancer.

## MATERIALS AND METHODS

### Clinical samples and mice

176 pairs of gastric cancer and para-cancer tissues were collected from patients in the First Affiliated Hospital of China Medical University [49]. All fresh resected tissues were washed clearly and dissected into small aliquots, and snap frozen in liquid nitrogen and store at -80°C. All patients or their guardians provided written informed consent, and this study was approved by the Ethics Committee of China Medical University. C57bl/6 p53^+/+^and p53^−/−^ mice were provided by professor Liu Cao in china medical university. 5 to 6 week old female BALB/c nude mice were purchased from Vital River Laboratory Animal Technology Co. Ltd (Beijing, China). All animal experiments were conducted in accordance with the Guide for the Care and Use of Laboratory Animals (NIH publication no.80–23, revised 1996) and approved by the Animal Ethic Committee of China Medical University.

### RNA isolation and real-time qPCR

Total RNA from cancer samples and cells were extracted by Trizol Reagent (Invitrogen, USA), then miRNAs were isolated with mirVana miRNA Isolation Kit (Ambion, USA). For detection of miRNAs, 500 ng microRNAs were reverse transcribed by PrimeScript™ RT Reagent kit (Takara, China) with specific primer. miR-135a RT primer:5′-GTCGTATCCAGTGCAGGGTCCGAGGTATTCGCACTGGATACGACTCACATAG-3′; U6 RT primer: 5′-AACGCTTCACGAATTTGCGT-3′. Then miR-135a expression was detected with SYBR^R^premix Ex Taq™ II kit (Takara, China) using the following primers: miR-135a forward, 5′-GCGCGTATGGCTTTTTATTCCT-3′, miR-135a reverse, 5′-CAGTGCAGGGTCCGAGGTC-3; U6 forward, 5′-CTCGCTTCGGCAGCACA-3′, U6 reverse, 5′-AACGCTTCACGAATTTGCGT-3′.

### Cell culture and transfection

Gastric cancer cell lines MGC-803, BGC-823, SGC-7901, MKN1, MKN45 and gastric mucosal epithelial cell line GES-1 were cultured in RPMI-1640 containing 10% FBS in a humidified 5% CO_2_ incubator at 37°C. Human umbilical vein endothelial (HUVEC) cells were cultured with ECM medium (ScienCell, USA). To generate MiR-135a overexpressing or Control BGC-823 and SGC-7901 stable cells, 1 × 10^4^cells were seeded into 12-well plates overnight. Then cells were infected with lentivirus-coated plasmids following the manufacturer's guideline (GeneChem, China), 5μg/ml puromycin (Sigma, USA) were added to select positive cells.

In order to overexpress p53, 2 × 10^5^ cells were seeded into 6-well plates, then 3μg pcDNA3.1flag-P53 or empty vector plasmids were transfected with Higene (Applygen, China) following the manufacturer's instruction. Cells were harvested 48 h after transfection, and total cell protein or RNA were extracted. For miRNA inhibitor transfection in transwell and tubule formation assay, cells are firstly transfected with 200 nM nonsense control RNA (NC) or miRNA inhibitor (200 nM) for 24 h, then treated with FAK inhibitor for 24 h prior to further experiments.

### Western blot assay

Cells were lysed with RIPA lysis buffer containing protease inhibitor (Roche, USA), then 30 μg total protein were subjected to 8% SDS-PAGE gel. Protein was transferred to a PVDF membrane (Millipore, USA) after separation. Membranes were blocked with 5% non-fat milk, and incubated with primary antibody overnight. FAK1 (Cell signaling technology, USA), ROCK1 antibody (Origene, USA), p-LIMK1 (Cell signaling technology, USA), GAPDH antibody (Kangchen, China), p53 (Santa Cruz, USA) and Flag (or DKK) antibody (Abmart, China) were used. Then appropriate secondary antibodies were added for 2 h at room temperature. Finally, protein were detected with enhance chemiluminescence (ECL) reagent (Thermo pierce, USA).

### Dual luciferase report assay

Primers used for pMIR-Report™ luciferase report vector were as the following: FAK-3′UTR-WT forward, 5′-AGACTAGTAGACCATTCCCCTCCTACCAG-3′, FAK -3′UTR-WT reverse, 5′-TTACGCGTA GGATATTACA ACAAGAACTTTACTGGT-3′; FAK-3′UTR-DEL (deletion) forward, 5′-CCATTTTTAT ATAATTTATAAAGGTT GAC TAT TTTACAGCCACTG-3′, FAK-3′UTR-DEL reverse, 5′-CAGTGGCTGTAAAA TAGTCAACCTTTATAAATTA TATAAAAATGGA-3′. 1 × 10^5^ cells were seeded into 24-well plate, then 200 ng luciferase report vector (WT/DEL), 10 ng pMIR-Report™ control plasmid and 100 nM miR-135a mimics or control mimics were co-transfected with Higene reagent. Medium was refreshed after 6 h and cells were cultured for 36 h. Finally, cells were lysed and the luciferase activity was measured using Dual Luciferase Reporter Assay System (Promega, USA). All experiments were conducted three times in triplicate.

### Clone formation

200 miR-135a overexpressing or control cells (2 groups) were seeded in matrigel (BD, USA) pre-coated 96-well plates and one control group added 10 μM FAK inhibitor (VS6063 also called Defactinib or PF04554878) (Sellckchem, USA). After 15 days, cells were photographed and counted by inverted microscope.

### 3-(4,5-Dimethylthiazol-2-yl)-2,5- diphenyltetrazolium bromide (MTT) assay

2 × 10^3^ miR-135a overexpressing or control cells were seeded into 96-wells and allowed to attach for 24 h. Then cells were incubated with 10 μL MTT (10 mg/mL) (Beyotime Biotechnology, China) at 37°C for 4 hours. Subsequently, medium was discarded and 200 μL DMSO was added into wells. After 20 min, the absorbance was measured at 490 nm with a microplate reader. Cell viability was measured for 7 days, and medium was refreshed every two days. All the experiments were repeated three times in triplicates.

### Apoptosis assay

1 × 10^5^ miR-135a overexpressing or control cells were seeded into 6-well plates. After 48 h incubation in 37°C and 5% CO_2_, cells were digested with trypsin and washed three times with cold PBS. Unfixed cells were stained with AnnexinV-PE and 7-amino-actinomycinD kit (Becton-Dickinson, USA) following the manufacture's instructions. Subsequently, cells were subjected to flow cytometric analysis with FACSCalibur (Becton-Dickinson, USA). For VS6063-treated group, drug was added 24 h prior to apoptosis analysis.

### Transwell assay

Transwell migration and invasion assays were performed with Transwell insert chambers (Corning, USA). 1 × 10^5^ cells were resuspended in 100 μL FBS serum free medium and seeded into the top chamber, while the medium containing 10% FBS was loaded into the bottom of chambers. After 24 hours, cells were fixed with 4% paraformaldehyde and stained with 0.4% crystal violet. Cells onto the upper chamber were removed with a cotton swab, washed and air-dried. Images of cells on the transwell membrane were taken with a microscope at 200× magnification. Then crystal violet was dissolved with 33% acetic acid, and the absorbance was measured with spectrophotometer at OD570. For invasion assay, the upper chambers were pre-coated with matrigel (1:10 diluted, BD, USA).

### Tubule formation assay

2 × 10^5^ miR-135a overexpressing or control cells (2 group) were seeded into 6-well plates, and one control group was treated with 10 μM FAK inhibitor VS6063. After 24 h, medium was refreshed, and cells were cultured for another 24 h. Afterwards, conditioned medium (CM) from these cells were centrifuged at 10000 rpm, and the supernatant was collected. For tubules formation, 8 × 10^3^ HUVEC cells were seeded into Matrigel pre-coated 96-well plates, then treated with 50μL conditioned medium for 6 h. Tubules were photographed by microscopy and counted by Image Pro Plus software. Experiments were performed in triplicate and repeated three times.

### Tumor bearing nude mice

1 × 10^6^ miR-135a overexpression and control tumor cells (pre-mixed with 50 μL matrigel before injecting) were injected into bilateral axillary of BALB/c nude mice (*n* = 6 per group). 30 days later, mice were executed, tumors samples were excised and tissues weight was measured with an electronic balance. After washing carefully with PBS for 3 times, tumor samples were fixed with 4% paraformaldehyde and embedded into paraffin block.

### Immunohistochemical and hematoxylin staining

Sections cut from paraffin-embedded nude mice tumor samples were deparaffinized and rehydrated. Immunohistochemical staining assay was used to detected the expression of CD34 in all tumor sections with CD34 antibody (1:250, Abcam, USA) and Elivision™ plus (Maixin Biotechnology Co. Ltd, China). Then cell nucleus was stained with hematoxylin (Maixin Biotechnology Co. Ltd, China). After hydration and transparent, sections were sealed with neutral resins and photographed. Finally, CD34 positive vessels which were defined as blood vessels were analyzed with Image Pro Plus.

### Chromatin immunoprecipitation (CHIP) assay

Logarithmically growing BGC-823 were seeded in the 10 cm plates (1 × 10^6^), then transfected with 15μg pcDNA3.1flag empty plasmid or pcDNA3.1flag-p53 plasmid. After 48 h, cells were fixed with 1% final concentration formaldehyde solution, followed by sonicating with ultrasonic cell crusher on ice. Cells supernatants were incubated with anti-Flag antibody, and immunoprecipitation with Protein-A beads (Merck Millipore, USA). The beads were then washed with buffer and eluted with elution buffer. Then cross-links were reversed at 65 overnight. The DNA was purified with DNA isolation kit (Axygen, USA) and eluted with TE buffer (10mM Tris-HCl, 1mM EDTA, PH = 8.0). PCR was conducted using the following primers: forward, 5′-CGCTTACATAGTCAAACAGGTACT-3′, reverse, 5′-TCAAGCCTCTTGCTCCAACT-3′.

### Bioinformatics analysis

Microarray data of GSE39358 was downloaded from GEO database. Differentially expressed miRNAs between parent cell lines and metastatic cell lines were analyzed with R package. Protein-protein interaction was predicted by STRING software. Potential target genes that interacted with miR-135a and their functional cluster were analyzed with StarBase software. The genomic status of BRCA1 and TP53 in gastric cancer were searched and analyzed with cBioPortal.

### Statistical analysis

The expression of miR-135a in gastric cancer tissues was estimated with one-sample *t* test. The association between miR-135a expression and clinical characters was analyzed with one-way ANOVA. The overall survival was analyzed using Kaplan-Meier method and log-rank test. Statistical significance between the control and experimental groups was determined using paired-samples *t* test. All analysis was performed by SPSS version 17.0, and data was expressed as mean ± SD. A *p value* of <0.05 was regarded as statistically significant.

## SUPPLEMENTARY MATERIALS FIGURES AND TABLES





## Abbreviations

FAK: Focal adhesion kinase
ROCK: Rho-associated protein kinase
LIMK: LIM domain kinase
BRCA1: Breast cancer 1
PCR: polymerase chain reaction
