# Supplementary material for: miR-135a inhibits tumor metastasis and angiogenesis by targeting FAK pathway
Source: Oncotarget. 2017 Mar 10;8(19):31153–68. doi: 10.18632/oncotarget.16098 (PMC5458197; doi:10.18632/oncotarget.16098)
Supplement: Supplementary file 1 [file oncotarget-08-31153-s001.pdf]

miR-135a inhibits tumor metastasis and angiogenesis by targeting FAK pathway

Supplementary Materials

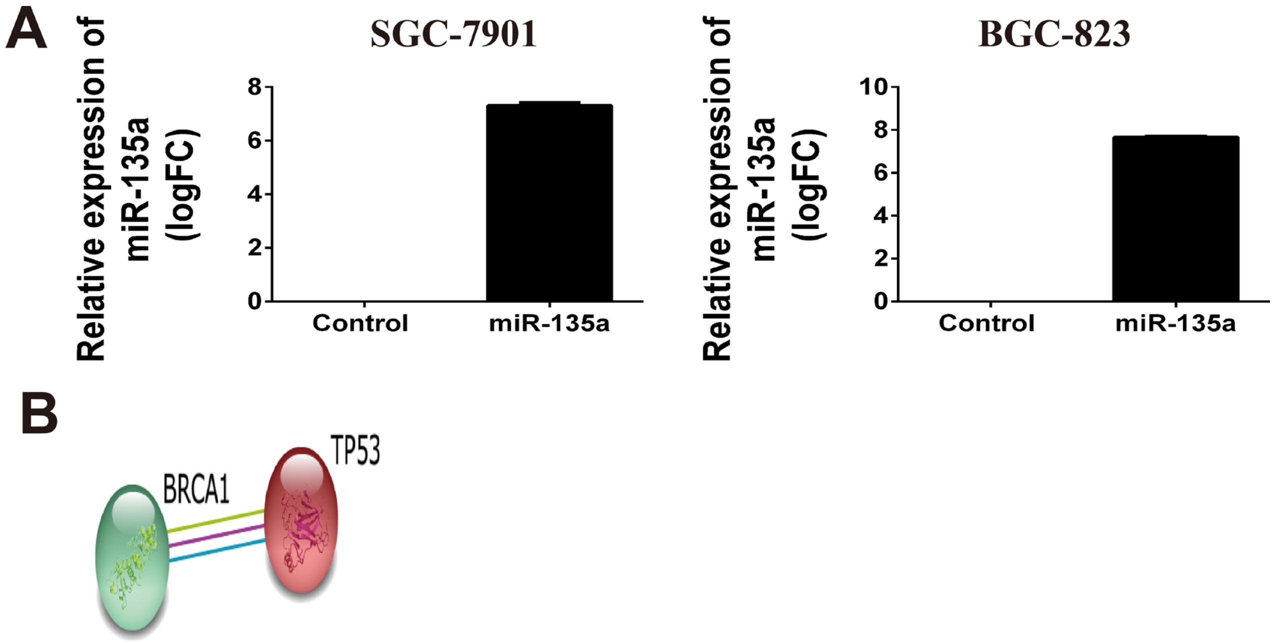

Supplementary Figure 1: (**A**) Relative expression of miR-135a in stable cell lines is examined by Real-time PCR. (**B**) The interaction between TP53 and BRCA1 is analyzed by STRING software.

Supplementary Table 1: The clinicopathological characteristics of gastric cancer patients

parameters	No.
Age(yrs)	
<65	113
≥65	63
Gender	
Male	131
Female	45
Family history	
NO	161
Yes	15
Histologic grade	
Well & Moderate	63
Poor & Signet	113
Size (maximal diameter)	
< 5cm	76
≥ 5cm	100
Histological type	
Intestinal	82
Diffuse	94
Depth of invasion (pT)	
T1, T2	35
T3, T4	141
Lymph node status (pN)	
N0, N1	84
N2, N3	92
Pathological stage (pStage)	
Stage I	21
Stage I,II,IV	155

Supplementary Table 2: Putative TFs were predicted with 75% threshold in 1000 bp upstream sequence of miR-135a-2. See Supplementary_Table_2
